# Development of Nitrogen Recycling Strategies for Bioregenerative Life Support Systems in Space

**DOI:** 10.3389/fmicb.2021.700810

**Published:** 2021-10-13

**Authors:** Tom Verbeelen, Natalie Leys, Ramon Ganigué, Felice Mastroleo

**Affiliations:** ^1^Microbiology Unit, Interdisciplinary Biosciences, Belgian Nuclear Research Centre (SCK CEN), Mol, Belgium; ^2^Center for Microbial Ecology and Technology (CMET), Faculty of Bioscience Engineering, Ghent University, Ghent, Belgium; ^3^Centre for Advanced Process Technology for Urban REsource Recovery (CAPTURE), Ghent, Belgium

**Keywords:** space exploration, bioregenerative life support systems, MELiSSA, urine recycling, nitrogen recovery, nitrification, ureolysis

## Abstract

To enable long-distance space travel, the development of a highly efficient and robust system to recover nutrients from waste streams is imperative. The inability of the current physicochemical-based environmental control and life support system (ECLSS) on the ISS to produce food *in situ* and to recover water and oxygen at high enough efficiencies results in the need for frequent resupply missions from Earth. Therefore, alternative strategies like biologically-based technologies called bioregenerative life support systems (BLSSs) are in development. These systems aim to combine biological and physicochemical processes, which enable *in situ* water, oxygen, and food production (through the highly efficient recovery of minerals from waste streams). Hence, minimalizing the need for external consumables. One of the BLSS initiatives is the European Space Agency’s (ESA) Micro-Ecological Life Support System Alternative (MELiSSA). It has been designed as a five-compartment bioengineered system able to produce fresh food and oxygen and to recycle water. As such, it could sustain the needs of a human crew for long-term space exploration missions. A prerequisite for the self-sufficient nature of MELiSSA is the highly efficient recovery of valuable minerals from waste streams. The produced nutrients can be used as a fertilizer for food production. In this review, we discuss the need to shift from the ECLSS to a BLSS, provide a summary of past and current BLSS programs and their unique approaches to nitrogen recovery and processing of urine waste streams. In addition, compartment III of the MELiSSA loop, which is responsible for nitrogen recovery, is reviewed in-depth. Finally, past, current, and future related ground and space demonstration and the space-related challenges for this technology are considered.

## Introduction

Space missions are primarily driven by the desire to acquire new knowledge in a wide variety of scientific fields, such as life sciences, physics, material science, and planetary science. Currently, all long-distance space missions to Mars, for example, are conducted by robots. While cheaper than a crewed mission, more accurate and reliable, these expeditions mainly serve as precursor missions. Humans can handle tasks far more complicated than those which can be performed with robotic automation. They bring versatility, adaptability, a hands-on approach, and problem solving skills to the table. All of which cannot be underestimated in distant and high-risk environments. However, due to the complicated nature of crewed missions over extended time periods, many new technologies and engineering processes still need to be developed (Crawford, 2004; Rovetto, 2016).

One of the greatest challenges of long-distance space travel and the establishment of bases beyond Earth’s orbit is the ability to provide food, water, and a breathable atmosphere for the crew in a stable and secure manner with a high reliability over time. It is estimated that the life support of a single crew member demands 1.83kg of food and 2.50kg of water per day (Anderson et al., 2018). Assuming a 3-year mission to Mars with a crew of four, a total payload of 25,287kg would be needed for food and consumable water alone. From a logistics perspective, carrying out such a mission by relying on periodic resupply missions with spacecrafts is challenging due to the large payloads and great distances from Earth. Also, prices of cargo to space are costly, currently exceeding $10,000 per kg which makes such an approach cost-prohibitive (Clauwaert et al., 2017; Pickett et al., 2020). Hence, minimization of transport costs is another incentive to reduce the payload size of any space mission.

*In situ,* production of vital resources tackles some of the aforementioned challenges and has already been implemented in space stations, such as Mir and the international space station (ISS) in the form of life support systems. In those technologies, water and air managements play an essential role in recovery of oxygen and potable water. The remaining waste is stored and destroyed upon re-entry in Earth’s atmosphere. On the ISS, the environmental control and life support systems (ECLSSs) are entirely based on physicochemical processes and are responsible for the production of potable water and oxygen with the help of waste stream recycling (Bagdigian et al., 2015; Volpin et al., 2020). As a result, the transport payload of water can be reduced by as much as 96.5% (Hendrickx et al., 2006; Clauwaert et al., 2017). However, the operation of the ECLSS requires a steady supply of consumables. Furthermore, nutrition has to be provided from terrestrial sources since it is not considered in current operative systems.

Bioregenerative life support systems (BLSS) are being developed as alternatives to existing fully physicochemical technologies. A BLSS aims at covering the metabolic needs of the crew by recovery of nutrients from waste streams in a closed-loop system through the combination of biological and physicochemical processes. For these systems, a minimal amount of consumables is needed, generated waste can be recycled, and food, water, and oxygen are produced. In most of the BLSSs, urine recycling plays an essential role in the recovery of water, but also in providing a nitrogen source for higher plant and/or edible bacteria biomass growth. With an average daily excretion of 7–16gN per crew member, urine accounts for 85% of the total potentially recoverable nitrogen in a BLSS, mostly under the form of urea. This makes urine the main source of nitrogen in these systems. Different strategies are applied to provide a suitable form of nitrogen for plants and micro-algae to assimilate, either by directly combining urine with a nutrient stream or indirectly through production of an appropriate fertilizer. Plants and micro-algae are cultivated with these nutrient streams and serve as an important source of the required dietary protein intake for crew members (Paradiso et al., 2014; Ilgrande et al., 2019a). Although biological systems that recover nitrogen are already operative in terrestrial settings, the space environment subjects organisms to microgravity and increased ionizing radiation intensities. These factors may affect the way microorganisms may behave, ultimately affecting the performance and stability of a BLSS. This should be taken into consideration when putting forward the nitrogen recovery space technologies development roadmap.

In this review, we describe the current ECLSS on the ISS and its limitations and summarize different BLSS systems and their urine treatment strategy. We also provide an in-depth report on the nitrogen recovery strategy of the Micro-Ecological Life Support System Alternative (MELiSSA) and its challenges for space travel. MELiSSA is the BLSS strategy of the European Space Agency (ESA) and is based on the ecosystem of a lake. It is subdivided in five compartments, each representing a subsection of a (closed-loop) lake ecosystem. The program has been under development for over 30years, making it the longest-running program out of all BLSS initiatives. Moreover, it was the first of its kind, approaching BLSS development from an engineering point of view. These two factors combined to make it one of the most advanced and promising concepts to support future exploration. Recent research on both improvements and developments of the MELiSSA nitrogen recovery technology as well as studies on the biological effects of real and simulated space conditions on nitrifying bacteria is highlighted here.

## Current Eclss on the International Space Station

**Figure 1:**
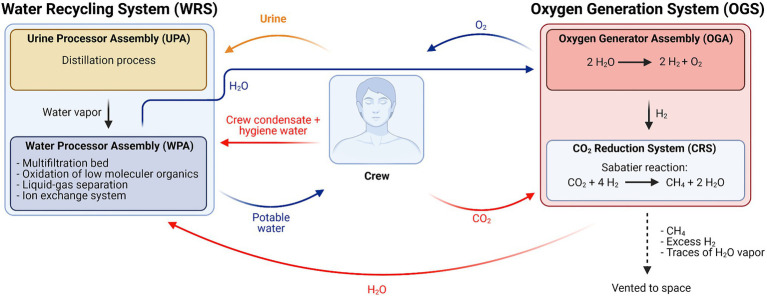
Simplified schematic overview of the Environmental Control and Life Support System (ECLSS) currently in use on the International Space Station (ISS; Adapted from Volpin et al., 2020).

ISS’s ECLSS is comprised of the water recovery system (WRS) and the oxygen generation system (OGS; Figure 1). Operational since 2007 and 2008, respectively, it integrates the different life support systems in different modules, whereas before this, each space agency provided its own life support system. The current ECLSSs have helped to meet the water and oxygen demands of astronauts through physicochemical processes (Bagdigian et al., 2015).

Oxygen is produced in two interconnected processes: (1) in the oxygen generation assembly (OGA), the water (H_2_O_liquid_) obtained from Earth supplies and (2) from the WRS, where H_2_O is electrolyzed to H_2_ and O_2_. The produced H_2_ reacts with metabolic CO_2_, originating from crew respiration and collected from cabin atmosphere, *via* the Sabatier reaction (eq. 1) in the CO_2_ reduction system (CRS). In this process, CH_4_ and H_2_O are generated (Greenwood et al., 2018).


(1) $${\mathrm{CO}}_{2}+4\;H_{2}\to {\mathrm{CH}}_{4}+2\;H_{2}O$$

Produced H_2_O is purified in the WRS, together with a water stream originating from processed urine from the Urine Processor Assembly (UPA), and returned to the OGA. CH_4_ is vented to space (Greenwood et al., 2018).

### Water Recovery System

**Figure 2:**
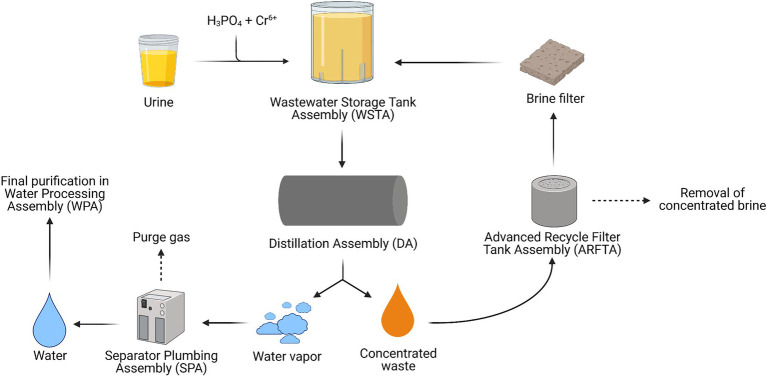
Schematic overview of the Urine Processor Assembly (UPA) module of the Water Recycling System (WRS) operating on the International Space Station (ISS; Adapted from Bagdigian et al., 2015).

The WRS can be subdivided into two assemblies operating in concert with each other. The Water Processor Assembly (WPA) collects condensate from the cabin atmosphere originating from crew perspiration and respiration, water from the OGS produced during the Sabatier reaction, and distillate from the second module, the UPA. The UPA’s position in the ECLSS is shown in Figure 1. In Figure 2, a schematic overview of the UPA module is provided (Bagdigian et al., 2015; Volpin et al., 2020).

In total, an average of 1.80l of urine (4–9gN/L d^−1^) and urine flush water per crew member per day is collected in the Wastewater Storage Tank Assembly (WSTA). Here, the urine load is kept sterile and is chemically stabilized to avoid scaling (precipitation of solid minerals in urine collection systems) by mixing it with an H_3_PO_4_ and Cr^6+^ solution. Originally, H_2_SO_4_ played the role of H_3_PO_4_ in the system, acidifying urine and converting the volatile ammonia (NH_3_) originating from the hydrolysis of urea to non-volatile ammonium (NH_4_^+^). However, astronaut’s urine contains higher amounts of Ca^2+^, which can precipitate as CaSO_4_ and cause scaling and pipeline clogging which eventually leads to system failure. This issue was initially addressed by reducing UPA water recovery from 85 to 75%, which prevented the CaSO_4_ concentration from reaching its solubility limit in water. The introduction of H_3_PO_4_ to acidify stored urine significantly reduced SO42-content and hence scaling potential. This allowed the increase of water recovery efficiency up to 85% again. Cr^6+^ acts as an oxidizing agent, preventing urea hydrolysis and thus avoiding NH_4_^+^ formation (Muirhead et al., 2018; Volpin et al., 2020). Chemically stabilized urine is pumped from the WSTA to the distillation assembly (DA) after enough urine has been collected in the storage tank (Bagdigian et al., 2015; Anderson et al., 2018; Volpin et al., 2020).

In the DA, water is separated from waste products through evaporation, leaving a concentrated waste mixture behind. This waste stream passes through the Advanced Recycle Filter Tank Assembly (ARFTA) where concentrated brine is collected with a series of membrane filters (Link et al., 2010; Bagdigian et al., 2015; Volpin et al., 2020). A downstream brine filter removes leftover solid precipitates formed during the process (Link et al., 2010). After every cycle, the ARFTA becomes saturated and is emptied and the removed concentrated brine is stored until eventual disposal. The resulting filtered waste stream is then returned to the DA for a new distillation cycle. Finally, the produced water vapor from the distillation process is separated from purge gases before passing to the WPA (Bagdigian et al., 2015; Volpin et al., 2020). In the WPA, water undergoes the final purification steps. Here, contaminants are removed in a four-step process: (1) removal of inorganics and non-volatile organic compounds in an ion-exchange resin multifiltration bed, (2) oxidation of low molecular organics with oxygen at 130°C, (3) liquid–gas separation of oxygen and other leftover gaseous by-products, and (4) removal of carbonate and bicarbonate ions through an ion-exchange system (Volpin et al., 2020).

### Limitations of the Current ECLSS

Although the current ECLSS is suitable for space missions in Low Earth Orbit (LEO), there are several limitations that need to be addressed to enable its use in long-term space missions. In order to make long-distance space exploration feasible, a life support system should recover more than 98% of water and nutrients from waste streams and produce food to meet the human metabolic needs, with minimal use of additional resources (Pickett et al., 2020). The UPA operating efficiency of only 85% results in a significant loss of available H_2_O. In addition, not all water vapor that can be used for oxygen production is condensed out of the CH_4_ waste stream in the OGS, thereby venting valuable water to space. The combination of the H_2_ lost under the form of CH_4_ (as H-atoms) and the loss of uncondensed H_2_O produced in the Sabatier reaction results in only 50% of O_2_ recovery produced with the Sabatier reaction from metabolic CO_2_ (Greenwood et al., 2018). Consequently, though designed for high reliability, the current ECLSS does not meet the necessary requirements for long-distance space travel. Water resupply from Earth is necessary to enable system operation. Moreover, 0.21kg of disposable hardware (saturated filters, malfunctioning hardware, etc.) is consumed per 1l of potable water produced, which need to be replaced during resupply (Volpin et al., 2020). In conclusion, while payload size and storage requirements on the ISS have been vastly reduced by the use of the ECLSS, they are still considerable and should be minimized further. Moreover, energy requirements to employ ECLSS modules are substantial (Jones et al., 2014). Hence, technological improvements (e.g., higher water recovery efficiencies, nutrient recovery, and higher system reliability) or a switch to new innovations, such as a BLSS, is required (Jones, 2016). The latter would allow tapping on waste streams rich in valuable compounds (N, P, and K), such as brine produced from urine during water purification and solid wastes, both of which are currently stored to be incinerated upon re-entry into Earth’s atmosphere. These compounds could be alternatively used for the production of fertilizer for food production (Volpin et al., 2020). It would help to (partially) solve the inability of the current system to produce food and reduce the need for frequent supply missions to space.

## Bioregenerative Life Support Systems

Bioregenerative life support systems are able to convert inorganic and organic waste into food, water, and oxygen through the combination of biological and physicochemical processes. *In situ,* production of a balanced diet for crew members during a long-distance space mission is currently only possible when incorporating biotechnological aspects into life support systems. Several projects were developed or are still in development at different major space organizations, and an overview of these projects is provided in the following paragraph. In the scope of this review, the unique urine processing systems of each of these technologies are emphasized (Table 1).

**Table 1:** Overview of different BLSS systems.

BLSS	Organization	Urine and nitrogen processing system	Bacterial consortium	Advantages	Disadvantages	Space experiments	Nitrogen recovery efficiency	References
BIOS-3	Institute of Biophysics of Siberian Branch of the Russian Academy of Sciences	Unprocessed urine mixed with a nutrient solution fed to higher plants	None	Simplicity of technology	Accumulation of NaCl in the inedible biomass of plants; nitrogen demands of cultivated wheat plants are not entirely met with urine alone	None	N/A	Lisovsky et al. (1997) and Salisbury et al. (1997)
Biosphere 2	Biosphere 2	Marsh biome system	Natural bacteria in Marsh biome	N/A	N/A	None	N/A	Guo et al. (2017)
Closed Ecological Experiment Facility (CEEF)	JAXA	Waste incineration	None	CO_2_ production for crop growth	High energy consumption; high temperatures for incineration; highly oxygen consuming process	None	N/A	Ashida and Nitta (1995) and Tako et al. (2017)
Lunar Palace 1	National Natural Science Foundation of China	Reduced-pressure distillation followed by treatment in a membrane aerated, activated carbon bioreactor	Populated naturally by microorganisms from Lunar Palace environment & wastewater	Potentially high degree of adaptability to space conditions	Loss of urea-nitrogen during distillation process	None	20.5 %	Xie et al. (2017)
Lunar Palace: In development		Aerobic membrane bioreactor and anaerobic membrane bioreactor	Sludge from municipal wastewater treatment plants	Potentially high degree of adaptability to space conditions; ability to recover nitrogen from urea	High energy costs	None	80 - 99 %	Cheng et al. (2019)
Closed Equilibrated Biological Aquatic System (C.E.B.A.S.)	DLR	Ammonia oxidizing biofilter	Bacteria of the *Nitrosomonas* and *Nitrobacter* genera	N/A	N/A	C.E.B.A.S MINI MODULE	N/A	Blüm et al. (2003)
Combined Regenerative Organic food Production (C.R.O.P.)	DLR	Biofiltration process (biological trickle filter)	Natural community of soil microorganisms including *Nitrosomonas* and *Nitrobacter*	Low energy consumption; easy handling; low maintenance; high degree of adaptability to space conditions; low space occupancy requirements	Depends on convection and sedimentation forces; adaption required to membrane aerated flow filters/artificial gravity system for space	Eu:CROPIS (malfunctioned)	66 - 87 %	Bornemann et al. (2015, 2018) and Hauslage et al. (2018)
Micro-Ecological Life Support System Alternative (MELiSSA)	ESA	Fixed-bed bioreactor	Defined nitrifying community of *N. europaea* and *N. winogradskyi*	Easier to characterize and model; acceptable degree of robustness to space conditions	Less robust than more diverse bacterial communities; requires pretreatment of organic urine compounds	BISTRO; NITRIMEL	50 - 100 %	Perez et al. (2004, 2005) and Cruvellier et al. (2017)
MELiSSA: In development		To be determined, currently tested in CSTR	Synthetic community with a urease-positive heterotrophic strain, *N. europaea*, and *N. winogradskyi*	Easier to characterize and model; ability to recover nitrogen from urea in synthetic and fresh real urine; acceptable degree of robustness to space conditions	Less robust than more diverse bacterial communities; Oxygen competitiveness between constituents remains challenging	URINIS A1 and A2 (to fly)	35 - 94 %	Christiaens et al. (2019a) and Ilgrande et al. (2019a)

### Bioregenerative Life Support Systems in History

Russia spearheaded the development of BLSSs with the development of the Hybrid Biosphere System (BIOS) program, which was a large scale confined analogue environment manned by humans. It used microalgae and higher plants to meet a person’s oxygen demand (BIOS-1 and BIOS-2) and later expanded to also include plant-based food production (BIOS-3). Here, unprocessed human urine (containing urea) was mixed directly with nutrient solutions to successfully cultivate higher plants hydroponically (Salisbury et al., 1997; Guo et al., 2017). Other similar projects are the Biosphere 2 project of NASA, the Closed Ecological Experiment Facility (CEEF) of the Japan Aerospace Exploration Agency (JAXA), and the Lunar Palace 1 program of the National Natural Science Foundation of China. These facilities were built with a focus on *in situ* cultivation of higher plants and/or animals for future Lunar or Martian bases (Ashida and Nitta, 1995; Guo et al., 2017; Xie et al., 2017). The Biosphere 2 consists of a 1.27ha structure of different biomes (marshland, rainforest, savannah, etc.). In the 1990s, it was used to study artificial closed ecology systems with a human crew. Human urine was deposited in the marshland biotope, where it was digested through natural processes and bioavailable nitrogen could be redirected toward the remaining biomes (Nelson et al., 1999; Guo et al., 2017). In the CEEF, JAXA chose to incinerate human fecal material, urine, and inedible crop waste in an effort to recover CO_2_ for crop production. No nitrogen recovery system was put in place (Tako et al., 2017). More recently, China completed a 106-day terrestrial manned mission in the Lunar Palace 1 facility. A BLSS was successfully used to support a crew of four with minimal external input of food, water, and oxygen for the duration of the trial (Xie et al., 2017; Cheng et al., 2019). During the experiment, nitrogen was recovered from urine with an efficiency of 20.5% through reduced-pressure distillation, where ammonia was distilled together with the water vapor. The distillate from this process was combined with kitchen and sanitary wastewater and processed in a membrane bioreactor followed by activated carbon treatment. The former is populated by bacteria (including nitrifying strains) originating from the Lunar Palace environment and wastewater stream (Xie et al., 2017). It is worth mentioning that this system design was mainly focused on pollutant removal rather than optimization of nutrient recovery. In the second phase of Lunar Palace, an anaerobic and aerobic membrane bioreactor will replace the current setup. In preliminary experiments preceding a new manned mission in Lunar Palace, successful ureolysis has been observed in the anaerobic reactor, while the ammonium removal efficiency in the aerobic reactor sits between 80 and 99% during stable operation (Cheng et al., 2019).

The German Aerospace Center (DLR) initiated two smaller scale BLSS research projects. In the 1990s and the 2000s, the Closed Equilibrated Biological Aquatic System (CEBAS) combined aquatic vertebrates and invertebrates with microalgae, aquatic plants and nitrifying bacteria in a closed-loop ecosystem. An ammonia oxidizing biofilter, which contains bacteria from the *Nitrosomonas* and *Nitrobacter* genera, processed the excreted ammonium from organisms in the aquatic animal tank (Blüm et al., 2003). The second, more recent Combined Regenerative Organic-food Production (C.R.O.P) project utilizes a biofiltration process with a natural bacterial community (including *Nitrosomonas* and *Nitrobacter*) that populates a trickle filter. In this setup, nitrogen can be recovered from synthetic human urine that carries ammonium as N-source. The microalgae species *Euglena gracilis* provides oxygen necessary for nitrification activity in this setup. Tomato plants are cultivated using the resulting nutrient stream from the trickle filter. Depending on the dilution of a synthetic urine matrix, a 66–87% nitrogen recovery efficiency was realized. Here, an undiluted feed negatively impacted the nitrate production rates, while 60 and 80% diluted urine seemed to be within the optimal range for nitrogen recovery (Bornemann et al., 2015, 2018; Hauslage et al., 2018).

### The Micro-Ecological Life Support System Alternative

European Space Agency initiated the ambitious MELiSSA program in the late 1980s. It is currently the longest-running BLSS program to date. Based on a lake ecosystem, MELiSSA aims to develop a closed-loop bioregenerative system using an engineering-based approach. Five interdependent compartments utilize a variety of specific microorganisms or higher plants to produce oxygen, water, and food from human metabolic waste (Hendrickx et al., 2006; Lasseur et al., 2010). In contrast to the previous described projects and facilities that focused on the development of a large analogue human test facility, the MELiSSA Pilot Plant, a testbed facility of the MELiSSA loop, is designed to sustain the respiration needs of a single person while also providing 20–40% of necessary nutrition (Alemany et al., 2019). Hence, it is not designed to be a closed full-scale testing facility that can fully support a complete crew. The plant has been operative since 2009. The MELiSSA program is the oldest and most advanced circular BLSS currently in development. At the same time, it serves as a pioneer for the terrestrial circular economy as it provides state-of-the-art technology that can also be applied on Earth (Paladini et al., 2021).

**Figure 3:**
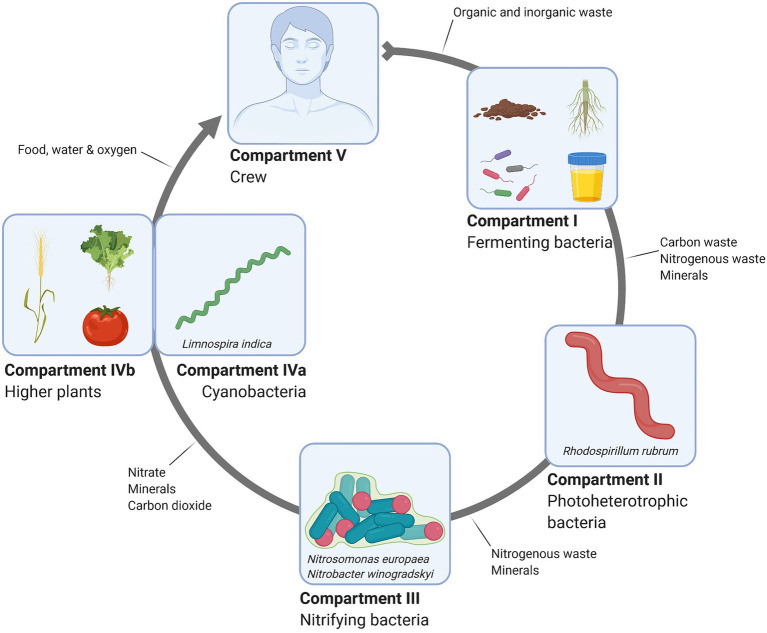
Schematic overview of the MELiSSA loop.

In the MELiSSA context, astronauts act as consumers, microorganisms are the recyclers, and plants and cyanobacteria close the loop as the producers (Figure 3; Lasseur et al., 1996; Hendrickx et al., 2006). In compartment I (CI), a consortium of bacteria digest/liquefy fecal and solid wastes and urine mainly into CO_2_, volatile fatty acids (VFAs), and minerals, including free NH_4_^+^. Bioreactor conditions are thermophilic and anaerobic to prevent possible pathogen proliferation and methanogenesis, respectively. Methane is a potentially hazardous and flammable biogas and hence it should be avoided on space vessels. Moreover, its potential use as a biogas fuel results in loss of carbon from the loop while the biological recovery of carbon from methane implies the introduction of two more bioreactors in the already highly complicated loop (Hendrickx et al., 2006; Hendrickx and Mergeay, 2007). Furthermore, carbon sources will also be lost as methane and recovering carbon from methane implicates two additional compartments, overcomplicating the MELiSSA design (Hendrickx et al., 2006). VFAs and minerals are further processed in Compartment II (CII) by the photoheterotrophic bacterium *Rhodospirillum rubrum* to produce biomass and additional NH_4_^+^ (Hendrickx et al., 2006; Mastroleo et al., 2009). Produced biomass could potentially serve as an additional nutrition source, while NH_4_^+^ is diverted to the third, nitrifying compartment (CIII; Hendrickx et al., 2006; Mastroleo et al., 2013). Moreover, removal of VFAs from the waste stream is necessary to ensure proper functioning of CIII, since its presence negatively affects nitrite oxidation in the dedicated compartment (Oguz et al., 2006; Mastroleo et al., 2013). Nitrogen availability is crucial for cyanobacteria (*Limnospira indica*) and higher plant growth in compartment CIVa and CIVb, respectively (Paradiso et al., 2014; Clauwaert et al., 2017; Alemany et al., 2019; Poughon et al., 2020). As such, to ensure optimal food production, an efficient nitrogen recovery system is indispensable. Although nitrogen fixation from urea by plants is possible, studies have shown that it is ineffective as plant fertilizer (Ilgrande et al., 2019a). Plants prefer to assimilate nitrogen from inorganic NH_4_^+^ and NO3-molecules while *L. indica* prefers NO3-over NH_4_^+^ and urea (Clauwaert et al., 2017; Ilgrande et al., 2019a). Therefore, in MELiSSA, urea is biologically converted to NH_4_^+^ and NO_3_^−^. In the context of a BLSS for space applications, NO3-is especially preferred due to the fact that NH_4_^+^ can easily convert to the volatile NH_3_, which is toxic at high concentrations in atmosphere. It can be harmful to the astronaut’s health if leaked into a closed environment, such as the ISS or any other spacecraft (Ilgrande et al., 2019a). Finally, NH_4_^+^ is also detrimental for plant growth at high concentrations (Paradiso et al., 2014; Ilgrande et al., 2019a). In CIII, NH_4_^+^ is oxidized to nitrate in a two-step process using O_2_ as electron acceptor. *Nitrosomonas europaea* is an ammonium-oxidizing bacterium that converts ammonium to nitrite, which is subsequently consumed by the nitrite oxidizing bacterium (NOB) *Nitrobacter winogradskyi*. Here, nitrogen is effectively converted to the form preferred by plants or cyanobacteria. It serves as a fertilizer in the subsequent biomass-and oxygen-producing and CO_2_-consuming compartment IV (Farges et al., 2012; Clauwaert et al., 2017; Ilgrande et al., 2019b). The final compartment V is the crew, who is consuming produced biomass and O_2_ on the one hand, and producing CO_2_, urine and fecal waste on the other hand. These waste products enter CI, closing the MELiSSA-loop.

In the MPP, bioreactors are operating and being characterized both individually and in an interconnected setup (Albiol et al., 2000; Gòdia et al., 2004). An important objective is to design associated technology that facilitates a continuous process that is robust and that runs as optimally as possible, as well as to develop mathematical models based on experimental data obtained from real operating conditions (Albiol et al., 2000).

## Nitrogen Recovery in CIII of Melissa

Traditionally, nitrifiers are responsible for ammonia nitrogen removal in wastewater treatment plants (Poughon et al., 2001; Farges et al., 2012). Autotrophic nitrifying bacteria have been proven to nitrify ammonium to nitrate in wastewater at rates which are orders of magnitude higher than those of heterotrophic bacteria (Perez et al., 2004). For nitrification of ammonium to nitrate, the MELiSSA system uses a synthetic community of two axenic autotrophic strains, i.e., *Nitrosomonas europaea* ATCC 19718 and *Nitrobacter winogradskyi* ATCC 25391, instead of a mixed undefined community from wastewater sludge (Cruvellier et al., 2016). An axenic co-culture does not perform significantly better than its open culture counterparts in a bioreactor (Zeghal et al., 1994), but it is easier to study, characterize, and model (Christiaens et al., 2019a). *N. winogradskyi* is the NOB considered in the MELISSA CIII because out of all NOB strain candidates studied, it proved to be the least affected by high concentrations of both ammonium and nitrite. This is a critical point in a system like CIII where ammonium load and, consequently, nitrite load can temporarily fluctuate to higher levels (Cruvellier et al., 2016). Biological activity at high efficiencies in highly saline environments is another prerequisite for nitrogen recovery from urine, where salinity can rise to 45–75 mS cm^−1^ in nitrified undiluted urine from 20 mS cm^−1^ in fresh urine (Christiaens et al., 2019a). A co-culture of *N. europaea* and *N. winogradskyi* was proven to tolerate salinities of up to 45 mS cm^−1^ in a synthetic urine matrix, working at nitrification efficiencies of 90–94% in a continuously stirred tank reactor (CSTR). The use of this co-culture could allow urine nitrification with limited dilution (Christiaens et al., 2019a).

### Nitrate Production With Nitrifying Bacteria

**Figure 4:**
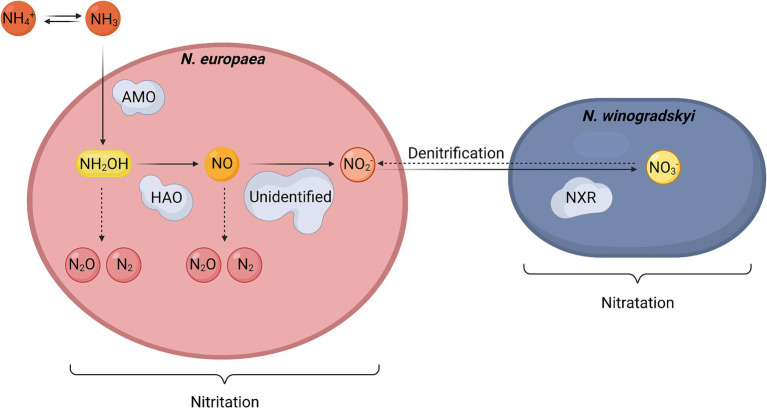
Simplified schematic representation of the nitrification pathway in an axenic co-culture of *Nitrosomonas europaea* and *Nitrobacter winogradskyi*. Full lines represent nitrification in aerobic conditions. Dashed lines represent the denitrification pathway in anaerobic conditions. Abbreviations: AMO: ammonia monooxygenase, HAO: hydroxylamine oxidoreductase, NXR: nitrite oxidoreductase.

The metabolic pathway of nitrification in *N. europaea* and *N. winogradskyi* is shown in Figure 4. Full nitrification is achieved by the nitrifiers in the following reactions (Cruvellier et al., 2016; Caranto and Lancaster, 2018):


(2) $${\mathrm{NH}}_{4}{}^{+}+1.5\;O_{2}\to {\mathrm{NO}}_{2}{}^{-}+2\;H^{+}+H_{2}O$$

(3) $${\mathrm{NO}}_{2}{}^{-}+0.5\;O_{2}\to {\mathrm{NO}}_{3}{}^{-}$$

The formation of nitrite from ammonium in *N. europaea* is called nitritation (1), and it is achieved in several enzymatic reactions. In the first step, ammonia is converted into hydroxylamine (NH_2_OH) by the membrane-bound ammonia monooxygenase (AMO). The next step involves hydroxylamine oxidoreductase (HAO), which was originally hypothesized to catalyze the oxidation of NH_2_OH to NO_2_- with O_2_ acting as the electron acceptor (Chain et al., 2003). Recently, however, nitric oxide (NO) has been demonstrated to be the enzymatic product of HAO instead of NO_2_^−^. Meanwhile, the production of NO_2_-from NO is probably catalyzed by a third, unidentified enzyme in the nitritation (Coleman and Lancaster, 2020). The presence of such an enzyme is necessary to outcompete the side reactions that can produce by-products, such as NO3-and/or N_2_O, but also to prevent the spontaneous formation of NO_2_^−^. The latter is required since non-enzymatic oxidation implies a loss of electrons, which are captured during the enzymatic reaction (Caranto and Lancaster, 2018). Finally, NO_2_-is oxidized to NO3-by the membrane-bound nitrite oxidoreductase (NXR) of *N. winogradskyi* during the so-called nitratation (2), also using O_2_ as an electron acceptor (Starkenburg et al., 2006; Cruvellier et al., 2016; Caranto and Lancaster, 2018; Coleman and Lancaster, 2020).

These nitritation and nitratation processes only take place in aerobic conditions since they are highly dependent on the oxidative power of O_2_. An anaerobic environment imposes a lack of oxygen on the cells for the nitrification reaction. In those situations, NO3-can be reduced to NO_2_-by *N. winogradskyi* using the NXR enzyme, which now acts as a reductase instead of an oxygenase under these circumstances (Freitag et al., 1987). *N. europaea* can further convert NO_2_-into gaseous N_2_O, NO, and N_2_ (Schmidt et al., 2004). This so-called nitrifier denitrification leads to nitrogen loss and production of undesired gasses. Following the requirements of an efficient and safe BLSS, denitrification should be strongly avoided.

### Characterization of the Fixed-Bed Bioreactor of the MELiSSA CIII

The nitrification reactor in the MPP is a fixed-bed bioreactor of 8.1l that houses a nitrifying co-culture of *N. europaea* and *N. winogradskyi*. It operates at a pH of 8.1 at 28±0.1°C and is magnetically stirred at the inlet to enable mixing of the bioreactor content (Montras et al., 2008). Due to the autotrophs’ characteristic low biomass growth and high conversion yield, the co-culture is grown as a biofilm immobilized on 4mm diameter polystyrene beads to avoid washout (Zeghal et al., 1994; Perez et al., 2004; Montras et al., 2008). Growth on biofilms allows decoupling of the liquid residence time in the reactor from the biomass retention time (critical for slow-growing organisms, such as nitrifiers), although substrate diffusion through biofilms can result in lower maximal conversion rates (Perez et al., 2004).

During an extended period of continuous operation (4.8years), nitrogen conversions in the order of 95–100% efficiency were reported in the MPP CIII, at ammonium loading rates of up to 1.35kgNm^−3^ d^−1^ (Gòdia et al., 2002; Perez et al., 2004; Montras et al., 2008). Despite the observed long-term stable operation of the bioreactor, it remains important to characterize the effects of certain perturbations on the process stability in the reactor. As mentioned, a critical parameter that influences nitrification efficiency is the dissolved oxygen (DO) availability in the reactor. Depending on how extensive oxygen limitation is, different steps in the nitrification process are affected (Gòdia et al., 2002). Hence, experiments were performed to quantify the bulk DO level needed in the system to ensure full nitrification (Zeghal et al., 1994; Gòdia et al., 2002; Perez et al., 2004). In a two-step decrease of DO concentration from 80 to 40% and 40 to 20%, ammonium was not completely converted to nitrite but all nitrite was oxidized to nitrate in the first step DO concentration decrease (80 to 40%). This indicates that DO concentration in this range is limiting for *N. europaea*, but not for *N. winogradskyi* (Gòdia et al., 2002). In the second DO decrease step (40 to 20%), however, ammonium was fully oxidized after an adaption period but nitrite conversion was incomplete. Here, *N. europaea* regained the upper hand in competition for oxygen with its co-culture counterpart and dominated the co-culture. These results showed that at 80% DO concentration, full nitrification was achieved while lower DO concentrations caused partial nitrification. It is thus essential to monitor DO in the bioreactor in order to control the nitrification process both in steady state and in dynamic situations (Gòdia et al., 2002).

Aside from high nitrification efficiency, another requirement for MELiSSA is stringent controllability of the compartments. MELISSA considers three control levels to ensure controllability (and thus stability) of the complex biological system: (1) real-time monitoring and control of predetermined parameters in each compartment (such as pH, temperature, and dissolved oxygen (DO) in the CIII), (2) implementation of a control law for each bioreactor chamber, and (3) an intercompartment coordination system for the loop (Gòdia et al., 2004). Specifically for the CIII compartment, several mathematical models have been constructed and validated to simulate and control the MPP CIII bioreactor in real-time. For instance, a hydrodynamic model able to predict the N-species response to ammonium load disturbances was developed, hence enabling the user to keep nitrite accumulation to a minimum (Perez et al., 2005). This is required due to the latter compound’s toxic properties for both plants and humans. Using the model, one can simulate the N-species (NH_4_^+^, NO_2_^−^, and NO_3_^−^) concentrations and biomass concentration in the fixed-bed bioreactor. The model serves as a valuable tool to maintain stable operation in CIII by identifying boundary conditions and operating the bioreactor within these boundaries (Perez et al., 2005). Beyond that, the heterogeneous distribution of the co-culture species was characterized along the length of the bioreactor after 4.8years of operation and this information was used to expand on the previous model to predict population dynamics and nitrification efficiency, while also considering diffusion of nutrients into the biofilm (Montras et al., 2008). Finally, Cruvellier et al. was able to determine that online measurements of base addition to counteracting nitritation-driven acidification (1) and online measurements of oxygen consumption can be used as predictive variables for partial nitrification (Cruvellier et al., 2016).

## Future Perspectives for Nitrogen Recovery

### Urea Hydrolysis: The Addition of Ureolytic Bacteria to the Nitrification Bioreactor

Nitrogen recovery from ammonium oxidation has been well characterized in ammonium-containing medium in the MELISSA CIII compartment at the MPP (Gòdia et al., 2004, 2004; Perez et al., 2015; Cruvellier et al., 2016, 2017). However, CIII cannot be used to directly process urine at present. Urine currently enters CI together with other waste products (Ilgrande et al., 2019b). Urea can be converted to ammonium by ureolytic bacteria through the action of the hydrolytic urease enzyme (Defoirdt et al., 2017; Ilgrande et al., 2019a). Urease catalyzes the conversion of urea into ammonium and carbamic acid. The latter then spontaneously hydrolyzes into a second ammonium molecule and carbonic acid, resulting in two ammonium molecules from one urea molecule and an increase in pH due to bicarbonate (HCO_3_^−^) formation (Defoirdt et al., 2017; De Paepe et al., 2020b).


(4) $$\mathrm{CO}{\left({\mathrm{NH}}_{2}\right)}_{2}+3\;H_{2}O\to 2\;{\mathrm{NH}}_{4}{}^{+}+{\mathrm{HCO}}_{3}{}^{-}+{\mathrm{OH}}^{-}$$

Full urine hydrolysis can take more than a month when relying on the spontaneous action of urease-positive bacteria populating urine pipelines and storage equipment. Storing urine for these extended periods of time is impractical for space applications (Defoirdt et al., 2017). By including an ureolytic heterotrophic bacterium directly in the nitrifying community in CIII, urea can be converted into ammonium (Defoirdt et al., 2017; Christiaens et al., 2019a; Ilgrande et al., 2019a). Low concentration organic compounds (~10g COD (Chemical Oxygen Demand) L^−1^) present in urine should also be removed from urine by the heterotroph to provide a fertilizer free of CODs, reducing the risk of biofouling of the nutrient solution for CIV (Christiaens et al., 2019a; Ilgrande et al., 2019a; De Paepe et al., 2020a).

Currently, research on adding one (or multiple) ureolytic strain(s) to the nitrifying co-culture in the CIII compartment is ongoing. The combination of a heterotroph with nitrifying bacteria in a defined community has not been demonstrated before, as opposed to wastewater treatment with mixed microbial communities. In the same study that demonstrated halotolerance of the nitrifying co-culture by Christiaens and co-workers, the co-culture was combined with three heterotrophic ureolytic strains (*Pseudomonas fluorescens* DSMZ 50090, *Acidovorax delafieldii* DSMZ 64, and *Delftia acidovorans* DSMZ 14801). Complete ureolysis and nitrification were achieved at similar efficiencies as a nitrifying co-culture (107±8% and 94±8%, respectively) when treating a 5% fresh real urine feed. When using a 10% urine influent, ureolysis and nitrification efficiencies significantly dropped to 66±9% and 35±3%, respectively. In another study by Ilgrande and coworkers, five ureolytic heterotrophic strains (*A. delafieldii, Comamonas testosteroni* I2, *Cupriavidus necator* DSMZ 13513, *D. acidovorans, P. fluorescens, Vibrio campbellii* LMG 22895) were also combined individually with the nitrifying co-culture. *C. necator* and *V. campbellii* containing communities did not show active nitrification of NH_3_. Meanwhile, ammonia oxidation rates in consortia with *P. fluorescens* and *C. testosteroni* were twice as high as those observed in *A. delafieldii* or *D. acidovorans* communities, indicating a beneficial interaction between the heterotroph and *N. europaea* (Ilgrande et al., 2019a). Moreover, in axenic conditions, *C. testosteroni, V. campbellii, and P. fluorescens* were proven to hydrolyze urea present in low concentrations, while the other strains (*A. delafieldii, C. necator,* and *D. acidovorans*) did not exhibit ureolytic activity here (Ilgrande et al., 2019a). Hence, the three former strains are favored over the latter three strains for possible inclusion in the nitrifying community since urea consumption will be more complete. Halotolerance of those ureolytic heterotrophs was also assessed and no ureolytic activity was observed for the selected species in high salinity (> 30 mS cm^−1^) conditions in an artificial urine matrix. However, ureolytic activity at lower salinities was not inhibited, implying the need for dilution of urine before feeding it to the bioreactor (Ilgrande et al., 2019a). Finally, organics removal was limited with all influent urine concentrations and has to be improved before space applications are possible (Christiaens et al., 2019a). Nonetheless, the inclusion of an ureolytic step in a nitrifying co-culture already shows successful ureolysis and nitrification, which is encouraging to the implementation of direct urine treatment in CIII.

### Stabilization of Fresh Urine for Long-Term Storage

Due to storage limitations in space, urine accumulation in a tank for extended periods of time is discouraged. However, storage cannot be avoided because urine is produced discontinuously over the course of a day. Unfortunately, untreated urine is highly unstable. As show in eq. 4, urea hydrolysis by urease-positive bacteria in the urine collection and processing system results in ammonium formation and a pH increase, causing ammonia (NH_3_) volatilization. This impacts nitrogen recovery and can have detrimental effects on crew members health. Moreover, salt precipitation due to high pH levels causing scale formation and clogging of the urine processing system (Coppens et al., 2016; De Paepe et al., 2020a,b). The toxic compounds (H_3_PO_4_ and Cr^6+^) that are used currently in the UPA system in ISS to stabilize stored urine can be replaced by a safer, electrochemical method (De Paepe et al., 2020b). Recirculation over a cathode in a three-compartment electrochemical cell is a potential alternative. Here, hydroxyl (OH^−^) ions are created from H_2_O and the pH is increased to 10–11 (due to hydroxyl-ion production), inhibiting enzymatic urease activity in a non-toxic manner (De Paepe et al., 2020b). Meanwhile, calcium and magnesium concentrations are reduced due to precipitation caused by supersaturation. This mineral removal process in the electrochemical cell also minimizes downstream clogging of the system (De Paepe et al., 2020b).

### Ureolysis and Nitrification in Space Conditions

**Figure 5:**
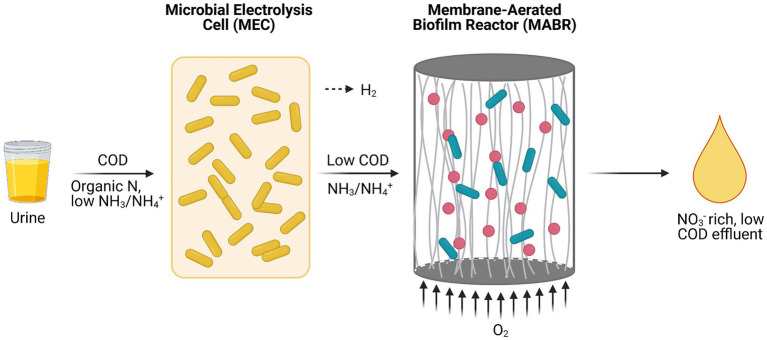
Schematic representation of a microbial electrolysis cell (MEC) and a membrane-aerated biofilm reactor (MABR) in a serial setup (Adapted from De Paepe et al., 2020a).

A key hurdle for nitrification in space applications is the diffusion of oxygen into the nitrifying culture due to reduced gravity conditions on Mars and microgravity conditions in LEO and deep space. Fluid dynamics are restricted to diffusion due to the absence of convection forces in these scenarios, causing limited oxygen distribution (Monti, 2001; Ilgrande et al., 2019a; De Paepe et al., 2020a; Acres et al., 2021). One possible way to circumvent this problem is the use of a membrane-aerated biofilm reactor (MABR) in which aeration takes place *via* gas-permeable membrane tubes populated by a biofilm of nitrifiers on the outer surface of the membranes. Since microorganisms directly take up the oxygen that diffuses through the membrane, no air bubbles are formed. However, bacteria closest to the gas-permeable membrane will consume most oxygen. This could be an issue if fast-growing ureolytic heterotrophs are present in the system, potentially limiting oxygen availability for nitrifiers on the outer layers of the biofilm. Additionally, anoxic areas may lead to denitrification by the autotrophic nitrifiers or by heterotrophic denitrifiers (using available COD) if an open community is used in the MABR. These denitrification reactions result in the loss of nitrogen as N_2_. COD removal with an ureolytic heterotroph in the reactor to minimize denitrification is too low. One option to tackle that issue while also preventing oxygen limitation for autotrophic nitrifiers is the addition of an upstream microbial electrolysis cell (MEC) populated by a ureolytic strain. The bacteria in the MEC remove organics efficiently using an anode as electron acceptor (Figure 5). Here, energy from COD is also partially converted to hydrogen gas. Ultimately, a low COD, nitrate-rich effluent suitable for CIV cyanobacteria and plant growth is obtained after treatment in a MEC-MABR setup (De Paepe et al., 2020a). The combination of physicochemical and biological treatment of urine optimizes nitrogen recovery at minimal energy input and high conversion efficiency while avoiding the use of toxic compounds for urine storage (De Paepe et al., 2020a,b).

Potential application of an electrochemical urine pretreatment module and a MEC-MABR module in the MELiSSA CIII shows promises, but still require modifications for operation in LEO. For example, hydrogen gas formed in the liquid in the MEC in space cannot escape due to the microgravity conditions. A possible solution is the use of a gas diffusion air cathode that prevents H_2_ formation in the MEC (De Paepe et al., 2020a).

While the former technologies are promising for improving the overall process of urine nitrification, the use of an anaerobic bioreactor followed by an aerobic bioreactor as will be applied in the Lunar Palace also show promise (Cheng et al., 2019). Thus, in recent years, exciting concepts have been demonstrated for the combination of physicochemical and biological elements for urine processing in space.

### Terrestrial Applications of Nitrogen Recovery Technologies

Several terrestrial applications based on space research for nitrogen treatment have been developed or are currently in development. For example, Biostyr™ is an innovative process that can be implemented to remove, among others, nitrogenous (NH_4_^+^, NO_3_^−^) compounds using both nitrification and denitrification in a compact structure, thereby presenting a low environmental footprint. Another technology based on a self-sustaining groundwater filtration system that removes nitrates with denitrification from the ground water to produce potable water has been installed and is currently in used in Morocco (MELiSSA Foundation, 2021). Finally, the Antarctica research facility Concordia has been fitted with a grey water processing system that includes biological components to remove nitrogen (MELiSSA Foundation, 2021).

As mentioned, untreated urine causes scaling and biofouling in downstream processes during urine recycling in CIII. This also holds true for pipelines in sewer and sewage plants. Stabilization of urine by controlled anaerobic ureolysis at the source rather than in a centralized manner can reduce divalent cation salt precipitation (Ca^2+^ and Mg^2+^) and release NH_3_ in a controlled manner. The latter is important to avoid nitrogen loss and thus improve downstream nutrient recovery, yielding environmental benefits as a consequence (Christiaens et al., 2019b). In a second potential system, an electrochemical cell drives precipitation of the divalent cation minerals at the toilet and, unlike the aforementioned technology, inhibits urea hydrolysis through alkalization of the urine. Source-separated urine can be stored and, when needed, discharged to a wastewater treatment plant that can recover nitrogen, reducing nitrogen loss (De Paepe et al., 2020b).

Hydrogen-oxidizing Bacteria (HOB) have been under the spotlight in recent years due to their high growth rate, low resource requirements, and a high protein content (> 70% of their cell dry weight). Hence, they have great potential to be used as microbial protein (MP) source (Christiaens et al., 2017; Yang et al., 2021). In recent studies, the growth of HOB on recycled nitrogen has been investigated (Christiaens et al., 2017; Yang et al., 2021). HOBs typically rely on ammonium as their nitrogen source and use H_2_ and O_2_ as electron donor and an electron acceptor, respectively, to fixate CO_2_ as carbon source (Yang et al., 2021). However, conventional ammonia removal and production with the Haber-Bosch process (an artificial industrial process to fixate nitrogen by converting atmospheric N_2_ to NH_3_ with hydrogen under high pressure and temperature) has a high energy cost. As a potential alternative, a proof-of-concept study validated the use of an electrochemical cell to extract NH_4_^+^ from hydrolyzed urine produced in, for example, a MEC. In the system, the urine is alkalified in a cathode compartment, which enables gas stripping of H_2_ and NH_3_. Then, the effluent is redirected to an anode compartment where NH_4_^+^ is pulled to the cathode compartment over an ion-exchange membrane. The O_2_ and H_2_ needed are generated at the anode and cathode, respectively, whereas the CO_2_ used as carbon source is a by-product of ureolysis. The use of this technology greatly reduces energy costs compared to NH_4_^+^ production with Haber-Bosch. A study on HOB species *C. necator* 335 confirmed its ability to grow at high NH_4_^+^ loads. Hence, it is possible to grow MP from HOB *via* NH_4_^+^ recovered from high NH_4_^+^ load waste streams such as urine (Yang et al., 2021). By supplementing human food or feed for cattle with MP from HOB produced from recovered nitrogen, nitrogen losses, energy costs, and surface area per unit of protein mass produced can be significantly reduced. This offers a strategy to tackle to challenges of future food production for an ever-growing human population (Christiaens et al., 2017; Yang et al., 2021).

## Impact of Space Conditions on Ureolytic and Nitrification Bacteria

In LEO, the metabolism of the ureolytic and nitrifying bacteria could be affected by increased ionizing radiation dose rates and changes in gravity conditions (Senatore et al., 2018; Milojevic and Weckwerth, 2020). Both spaceflight experiments and simulations on Earth have been conducted to observe and gain a broad understanding of the bacterial responses and, more importantly, to assess the feasibility of a BLSS for space exploration (Leys et al., 2009; Mastroleo et al., 2009, 2013; Lindeboom et al., 2018; Ilgrande et al., 2019b; Poughon et al., 2020; Senatore et al., 2020). It is, however, extremely challenging to perform flight experiments to assess the biological effects of a real space environment due to the high costs and great interest in flying an experiment in combination with limited crew time and space aboard the ISS and other space stations. Hence, terrestrial methods have been favored to simulate microgravity and increased ionizing radiation intensities to assess their effects in a more accessible manner.

### Bacterial Response to Simulated Microgravity

Depending on the location in spaceflight, microgravity decreases from 10^−3^ to 10^−6^ times the terrestrial gravity (Huang et al., 2018). These conditions indirectly impact bacterial life through the alteration of fluid mechanics. Buoyancy due to density differences, convection and hydrostatic forces are eliminated, causing a low-shear fluid environment for bacteria, in which nutrients and metabolites only spread by diffusion along a concentration gradient. This results in the formation of nutrient-depleted and metabolite-enriched zones around bacteria (Monti, 2001; Huang et al., 2018; Senatore et al., 2018; Acres et al., 2021). Microbial behavior in real microgravity conditions has been analyzed on the orbital stations Mir and ISS. Most experiments, however, have been conducted on Earth, using different variations of devices called clinostats, which simulate microgravity. The rotating wall vessel (RWV) is a type of 2D clinostat that rotates perpendicular to the gravity vector, causing low-shear fluid conditions and preventing the microbe from adapting to a specific gravitational orientation. These factors result in a low shear-modelled microgravity (LSMMG) environment (Orsini et al., 2017; Huang et al., 2018; Senatore et al., 2018; Acres et al., 2021). Alternatively, the random positioning machine (RPM), a type of 3D clinostat, changes the position of an experiment randomly at arbitrary speeds and directions in the 3D space. In doing so, the object in suspension experiences a net gravity vector close to zero over a certain time interval. For microorganisms, the RWV is the most commonly used device for experiments in simulated microgravity, while the RPM is generally applied for experiments with higher organisms, but several applications for bacterial experiments are also known (Mastroleo et al., 2009, 2013; Huang et al., 2018; Senatore et al., 2018, 2020; Acres et al., 2021). The effects of an LSMMG environment have been assessed for a wide variety of microbial species and have been observed to impact cell growth, cell morphology, cell metabolism, cell–cell communication, cell pigmentation, biofilm formation, stress response, gene expression, virulence, genetic transfer, and even cause genomic changes (Mastroleo et al., 2009, 2013; Orsini et al., 2017; Tirumalai et al., 2017; Senatore et al., 2020; Acres et al., 2021).

Very limited work has been performed on ureolytic and nitrifying bacteria or microbial consortia in simulated microgravity. Ilgrande and co-workers exposed a synthetic community of *C. testosteroni, N. europaea,* and *N. winogradskyi* to LSMMG in a 2D clinorotation experiment, showing similar ureolytic and nitritation activity in regular cultures compared to those subjected to microgravity (Ilgrande et al., 2019a). However, nitrite accumulated in the LSMMG-exposed samples, indicating inactive nitratation by *N. winogradskyi*. This observation has been attributed to the experiment setup, which was performed in a fluid processing apparatus used in former spaceflight experiments (Ilgrande et al., 2019a). It is hypothesized that this device could limit oxygen availability. The resulting competition for oxygen probably inhibited *N. winogradskyi* in the nitrifying community, likely causing nitrite accumulation in this scenario (Laanbroek and Gerards, 1993; Ilgrande et al., 2019a). Further work should ensure that the setup allows adequate oxygen availability for all members of the synthetic community. Moreover, an omics approach will aid in unraveling the underlying molecular mechanisms and constructing a thorough understanding of the behavior of nitrifying consortium in simulated microgravity conditions.

### Bacterial Response to Ionizing Radiation

A second major difference in the space environment compared to the terrestrial environment is the chronic low-dose ionizing radiation exposure. In LEO, radiation dose rates (400–600 μGy d^−1^) can be 150–200 times higher than those experienced on Earth (2–4 μGy d^−1^; Vanhavere et al., 2008; Mastroleo et al., 2009; Dachev et al., 2015). The average ionizing radiation exposure in lunar orbit was measured at 200–300 μGy d^−1^ and recent lunar surface measurements determined an average absorbed dose rate of 316.8 μGy d^−1^ (Reitz et al., 2012; Zhang et al., 2020). Finally, radiation doses in Mars transit are estimated to average 460 μGy d^−1^ while the Mars surface dose rate is approximated at an average of 210 μGy d^−1^ (Durante, 2014). Biological effects of ionizing radiation result from both direct and indirect damage to biomolecules. First, molecular bonds in biomolecules can be broken directly by radiation, which interacts with molecules by excitation and ionization. High energy, high density (HZE) particles are the main contributors to these effects. However, ionizing radiation mainly causes indirect damage by interacting with H_2_O molecules, creating reactive oxygen species that attack biomolecules and cause oxidative stress (Dartnell et al., 2008; Moeller et al., 2010; Hassler et al., 2014; Siasou et al., 2017).

There have been many studies on the effects of ionizing radiation on bacterial species. Cell survival to acute high-dose exposure is suggested to not rely on just a single mechanism, such as the ability to repair DNA, but rather a complex set of cellular responses. A review on studies of the effects on bacterial populations exposed to long-term chronic low doses in the Chernobyl restricted zone describes that radioresistance seems to improve (Mollert and Mousseau, 2016). This implies adaption of bacteria to long-term chronic low-dose radiation exposure. However, more rigorous experimentation is required to solidify this claim (Mollert and Mousseau, 2016). In experiments with *E. coli*, selective pressure caused by high-dose acute ionizing radiation (1,000Gy) led to highly radioresistant phenotypes (Byrne et al., 2014). The findings in the discussed experiments do imply the presence of selective pressure when bacteria are exposed to either chronic low-dose irradiation or acute high-dose irradiation.

Effects of space relevant doses of ionizing radiation mimicking LEO conditions have also been studied. It is impossible to precisely imitate the complex radiation environment on board the ISS due to the wide array of radiation types stemming from secondary particles generated from interactions of primary particles with the spacecraft’s hull. HZE particles are the main protagonists in radiobiological damage in space. Hence, irradiation with these types of particles in terrestrial simulation can give a close approximation of the biological effects of cosmic radiation (Moeller et al., 2010). Moreover, to more closely mimic the radiation environment in space, one could irradiate biological samples with both low-and high-energy particles. An experiment on *R. rubrum* (a MELiSSA CII strain) comparing the impact of simulated LEO ionizing radiation and a spaceflight experiment in ISS, showed a pronounced transcriptomic response to the radiation exposure. *R. rubrum* is thus able to respond and cope with conditions linked to spaceflight in LEO (Mastroleo et al., 2009).

To our knowledge, no research has been done on the effects of ionizing radiation on ureolytic and nitrifying strains or consortia. Only one study indicates that members of the ammonium-oxidizing *Nitrosomonadacaea* family, of which *N. europaea* is a member, are more sensitive to γ-irradiation than other soil microorganisms, and post-exposure recovery proceeds slowly (Shah et al., 2013). To provide an idea of the performance of the axenic ureolytic heterotrophs and nitrifying autotrophs in space, it is necessary to evaluate the biological effects of chronic low-dose ionizing radiation exposure on these strains. Moreover, valuable information can be gathered on radioresistance of these bacteria with an acute high-dose irradiation survival experiment.

### Bacterial Response to Spaceflight

Several studies have already provided researchers with insight into the effects of the LEO space environment on bacteria. Since it is hard to mimic the exact conditions of real space ionizing radiation and microgravity conditions and the combination of both, space experiments provide very valuable information of bacterial response to real space conditions. Such effects include variations in cellular metabolism, microbial proliferation rate, cell division, biofilm formation, cell morphology, cell motility, and genetic transfer between cells. Limited information is available on molecular responses of bacteria in space (Senatore et al., 2018; Milojevic and Weckwerth, 2020).

Nitrification in space has already been demonstrated in biofilters of the CEBAS experiment during the SPS-89 mission of the Columbia space shuttle (Blüm et al., 2003). A second biofilter experiment called C.R.O.P. failed due to hardware malfunctioning during spaceflight (Hauslage et al., 2018). Furthermore, two spaceflight experiments (NITRIMEL and BISTRO) have been conducted to specifically study the effects of space conditions on nitrifying bacteria and consortia. Both focused on the storage and survival of inactivated cultures of nitrifiers, rather than assessing the effects of space conditions on active cultures and the active nitrogen conversion process (Lindeboom et al., 2018; Ilgrande et al., 2019b). The impact of space conditions on reactivation ability of the nitrogen cycle cultures is important in case of system failure during spaceflight (Lindeboom et al., 2018). During the NITRIMEL experiment, reactivation of pure cultures of ureolytic heterotroph *Cupriavidus pinatubonensis, N. europaea,* and *N. winogradskyi*, a bipartite culture of *N. europaea* and *N. winogradskyi,* a synthetic consortium of all 3 strains, and a nitrifying bioreactor culture were assessed after a 44-day mission in LEO on board the FOTON-M4 research satellite. Samples were exposed to 547–827 μGy d^−1^ and microgravity (10^–3 _^ 10^−4^g) conditions. No impact on ureolysis, nitritation or nitratation rates was observed compared to control cultures stored on the ground (Lindeboom et al., 2018). These results were substantiated by the BISTRO fight experiment where *C. pinatubonensis,* autotrophic nitrifying *Nitrosomonas ureae, N. europaea, N. winogradskyi*, the bipartite community of *N. europaea* and *N. winogradskyi*, and a synthetic consortium of the aforementioned strains with or without *N. ureae* added to the community, were sent to the ISS for 7days. Here, the radiation exposure was 400 μGy d^−1^. A general biomass decay was observed for all ISS cultures as opposed to control samples, but reactivation of nitrifying processes was successful in all populations (Ilgrande et al., 2019b). The observations in both studies conclude that ureolytic and nitrifying strains can cope with exposure to space conditions for a duration of missions that can take us to at least the Moon and encourage further experimentation toward their application in a BLSS for space travel (Lindeboom et al., 2018; Ilgrande et al., 2019b).

In the current URINIS (Urine Nitrification in Space) project, the impact of spaceflight conditions on actively growing nitrification cultures in space will be assessed for the first time. A batch experiment followed by a bioreactor experiment will be flown to ISS to assess the *in situ* effect of space conditions on the nitrifying activity of pure strains and the synthetic consortium of *C. testosteroni, N. europaea,* and *N. winogradskyi*. Activity tests, viability tests, transcriptome, metabolome, and proteome analysis, phenotypical characterization and biofilm analyses will aid in understanding microbial responses to that environment and provide more answers for the feasibility of CIII in space travel.

## Conclusion

Bioregenerative life support systems will play an important role for both terrestrial and space applications in the future. In space, regenerative systems will be required for long-term space expeditions and colonization missions of celestial bodies. A closed loop system can meet the metabolic needs of the crew (food, water, and oxygen) where nitrogen recovery plays a vital part. Extensive research on urine recycling systems has enabled the development of several experimental technologies for which proof-of-concept experiments have validated their potential. However, the harsh space conditions and the stringent requirements for space applications require more research and development to further minimize energy costs, crew time, and nitrogen losses and to optimize efficiencies and continuous runtimes of the bioreactor process and robustness of the biological systems. Resource exploitation without adequate regeneration and/or recycling of waste results in overconsumption, pollution and resource scarcity. As such, recovery of valuable compounds from waste products could partly solve many environmental as well as societal issues (Pearson, 2007). Technologies obtained in the development of BLSSs for space travel show high potential for transfer to the resource recovery industry on Earth.

## Author Contributions

TV and FM collected literature and wrote the original draft. All authors critically reviewed the manuscript and figures and approved the final manuscript.

## Funding

This work was funded by the Belgian Federal Science Policy Office (BELSPO; Contract # PEA 4000129030) and ESA *via* the PRODEX program. The URINIS-A project is part of the MELiSSA program of ESA.

## Conflict of Interest

The authors declare that the research was conducted in the absence of any commercial or financial relationships that could be construed as a potential conflict of interest.

## Publisher’s Note

All claims expressed in this article are solely those of the authors and do not necessarily represent those of their affiliated organizations, or those of the publisher, the editors and the reviewers. Any product that may be evaluated in this article, or claim that may be made by its manufacturer, is not guaranteed or endorsed by the publisher.
